# Deciphering the combination mechanisms of Gualou–Xiebai herb pair against atherosclerosis by network pharmacology and HPLC-Q-TOF-MS technology

**DOI:** 10.3389/fphar.2022.941400

**Published:** 2022-09-01

**Authors:** Yarong Liu, Hua Zhong, Pengbo Xu, An Zhou, Lidan Ding, Jingwen Qiu, Hongfei Wu, Min Dai

**Affiliations:** ^1^ School of Pharmacy, Anhui University of Chinese Medicine, Hefei, China; ^2^ Anhui Province Key Laboratory of Research and Development of Chinese Medicine, Hefei, China; ^3^ The Experimental Research Center, Anhui University of Chinese Medicine, Hefei, China

**Keywords:** Gualou–Xiebai, herb pair, combination mechanisms, atherosclerosis, network pharmacology

## Abstract

**Introduction:** Gualou (*Trichosanthes kirilowii* Maxim)–Xiebai (*Allium macrostemon* Bunge) (GLXB) is a well-known herb pair against atherosclerosis (AS). However, the combination mechanisms of GLXB herb pair against AS remain unclear.

**Objective:** To compare the difference in efficacy between GLXB herb pair and the single herbs and to explore the combination mechanisms of GLXB against AS in terms of compounds, targets, and signaling pathways.

**Methods:** The combined effects of GLXB were evaluated in AS mice. The main compounds of GLXB were identified via quadrupole time-of-flight tandem mass spectrometry (UPLC-Q-TOF-MS) and UNIFI informatics platforms. The united mechanisms of GLXB in terms of nodes, key interactions, and functional clusters were realized by network pharmacology. At last, the anti-atherosclerotic mechanisms of GLXB were validated using enzyme-linked immunosorbent assay (ELISA) and Western blot in AS mice.

**Results:** The anti-atherosclerotic effects of the GLXB herb pair (6 g/kg) were more significant than those of Gualou (4 g/kg) and Xiebai (2 g/kg) alone. From the GLXB herb pair, 48 main components were identified. In addition, the GLXB herb pair handled more anti-atherosclerotic targets and more signaling pathways than Gualou or Xiebai alone, whereas 10 key targets of GLXB were found using topological analysis. Furthermore, the GLXB herb pair (6 g/kg) could suppress the inflammatory target levels of IL-6, IL-1β, TNF-α, ALOX5, PTGS2, and p-p38 in AS mice. GLXB herb pair (6 g/kg) could also ameliorate endothelial growth and function by regulating the levels of VEGFA, eNOS, p-AKT, VCAM-1, and ICAM-1 and reducing macrophage adhesion to vascular wall in AS mice. GLXB herb pair (6 g/kg) could improve the blood lipid levels in AS mice. In addition, the regulating effects of GLXB herb pair (6 g/kg) on levels of IL-1β, TNF-α, ALOX5, VEGFA, eNOS, VCAM-1, ICAM-1, and blood lipids were more significant than those of Gualou (4 g/kg) or Xiebai alone (2 g/kg).

**Conclusion:** The combination mechanisms of the GLXB herb pair were elucidated in terms of components, targets, and signaling pathways, which may be related to suppressing inflammation, regulating vascular endothelial growth/function, and improving blood lipid levels.

## GRAPHICAL ABSTRACT

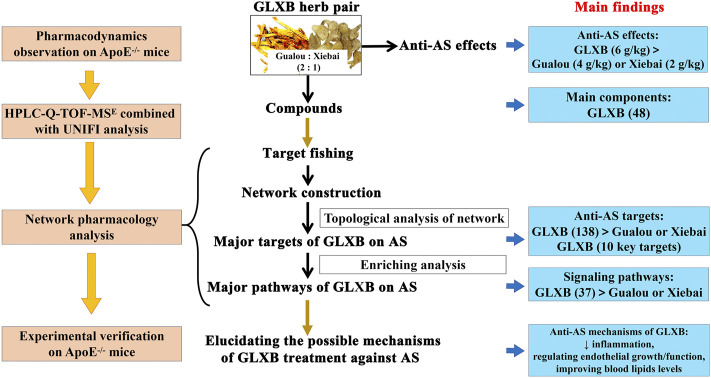

## 1 Introduction

Atherosclerosis (AS) is proven to be a lipid–driven inflammatory disease of the arterial intima, which is initiated by vascular endothelial injury and dysfunction (Back et al., 2019; Lacy et al., 2019). There are a number of genetic, metabolic, and environmental factors involved in the formation and evolution of atherosclerotic plaques (Zhu et al., 2018), which are characterized by the thickening, hardening, and reduced elasticity of large and medium arterial walls (Back et al., 2019). Single-target medicines usually fail as a treatment for this multifactorial disease. In addition, excessive or long-term use of these medicines may also develop resistance (Duan et al., 2020). Therefore, it is necessary and urgent to discover some new multitarget drugs.

GLXB herb pair consists of the dried fruit of *Trichosanthes kirilowii* Maxim (Chinese name Gualou) and the dried bulbs of *Allium macrostemon* Bunge (Chinese name Xiebai) in a weight ratio of 2:1 (Ding et al., 2016). The combination use of Gualou and Xiebai with the ratio of 2:1 was first proposed by Zhang Zhongjing (in the Eastern Han Dynasty, third century China) in the treatise “Jin Kui Yao Lue,” which was verified as the best ratio after a long period of clinical application (Zhang W. Y. et al., 2019). GLXB herb pair has been used clinically to treat cardiovascular diseases for thousands of years in China (Ding et al., 2013; Lin et al., 2018; Zhang W. Y. et al., 2019). In our previous studies, GLXB could significantly reduce aortic plaque formation and regulate metabolic disorders in ApoE^−/−^ mice (Xu et al., 2021). In addition, GLXB could also be used as a preventive drug for hyperlipidemia (Zhong et al., 2020). However, the difference in efficacy between the GLXB herb pair and the single herbs remains unclear. Furthermore, the combination mechanisms of GLXB herb pair against AS are worthy of study.

High-performance liquid chromatography coupled with quadrupole time-of-flight tandem mass spectrometry (HPLC-Q-TOF-MS) is a common qualitative and quantitative analysis technology, which is widely used to analyze and identify the structures of complex substances in herbal medicines (Xie et al., 2019). In addition, network pharmacology has become a new field of pharmacological study over the past few years (Zhang R. et al., 2019). An increasing number of studies explored the interactive association between the multicomponents, multitargets, and multipathways of the active herbal ingredients using network pharmacology (Zhou et al., 2020). In this study, the main compounds of GLXB and the single herbs were identified using HPLC-Q-TOF-MS and then ultimately determined by comparisons with the UNIFI informatics platform. Thereafter, a network pharmacology approach was used to reveal the combination mechanisms of GLXB for anti-atherosclerotic.

In our current study, we aimed to compare the difference in efficacy between the GLXB herb pair and the single herbs. Furthermore, we tried to explore the combination mechanisms of GLXB against AS in terms of compounds, targets, and signaling pathways. Altogether, our study innovatively provided a method for clarifying the advantage of the combination use of traditional Chinese medicine (TCM) in treating diseases.

## 2 Materials and methods

### 2.1 Ethics statement

Ethics approval and consent to participate in all animal experiments were approved by the Committee on the Ethics of Animal Experiments of Anhui University of Chinese Medicine (Ethics Statement number: AHUCM-mouse-2020018). International rules were strictly followed in handling the animals.

### 2.2 Reagents

High-grade Gualou and Xiebai were purchased from Beijing Tongrentang Co., Ltd. (batch number 181001, 190102), and confirmed by Professor Rongchun Han in the pharmacy department of Anhui University of Chinese Medicine. The voucher specimens were deposited in the Chinese medicine herbarium of Anhui Province Key Laboratory of Research & Development of Chinese medicine (Hefei, China).

Standard substances adenosine (YJ-110879) and rutin (YJ-100080) were purchased from National Institutes for Food and Drug Control (Beijing, China). ELISA kits of TNF-α (MM-0132M1), IL-1β (MM-0040M1), and IL-6 (MM-0169M1) were obtained from Enzyme Immune Industrial Co., Ltd. (Jiangsu, China); and eNOS (JL20482), ALOX5 (JL35097), and VEGF (JL12038) were obtained from Jianglai Biotechnology Co., Ltd. (Shanghai, China). ELISA kit of PTGS2 (M0755) was purchased from OSD Life Sciences Inc. (Shanghai, China). Kits of TC (A111-1-1), TG (A110-1-1), LDL-C (A113-1-1), and HDL-C (A112-1-1) were purchased from Jiancheng Bioengineering Institute (Nanjing, China). The antibody against GAPDH (A19056) was obtained from Beyotime Biotechnology (Shanghai, China), and the antibodies against p38 (AF6456), p-p38 (AF0211), AKT (AF6261), and p-AKT (AF0016) were obtained from Affinity Biosciences (Jiangsu, China). Antibodies against β-actin (ab8227), ICAM-1 (ab222736), VCAM-1 (ab174279), CD68 (ab125212), and α-SMA (ab5694) were purchased from Abcam Co., Ltd. (United States). Alexa Fluor-488 (SY0683) and Alexa Fluor-594 (SY0673) labeled secondary antibodies were purchased from Invitrogen (United States).

### 2.3 Preparation of herbs extracts

Gualou and Xiebai were pulverized into coarse powder before use. Gualou, Xiebai, and GLXB (2:1, w/w) were respectively immersed in 50% ethanol (1:5, w/v) for 1 h, and extracted twice with 50% ethanol for 2 h, referring to clinical application of GLXB herb pair (Ding et al., 2016). Later, the solution was concentrated to dryness on a rotary vacuum evaporator, freeze-dried, and then stored in a vacuum desiccator before use. The drug-extract ratio was 32.2, 25.6, and 12.6% respectively.

To assure the quality of herb extracts, fingerprinting was analyzed using HPLC. Adenosine and rutin were identified as chemical markers for quality monitoring, which had high quantity and obvious pharmacological activity in extracts of TCM (Riksen et al., 2008; Li et al., 2018). The conditions of chromatographic analysis and the representative HPLC were shown in the supplementary material.

### 2.4 Animals experimental design

Male C57BL/6 mice and ApoE^−/−^ mice (both were 7 ± 1 week in age, 22 ± 2 g in weight) were obtained from Changzhou Cavins Experimental Animal Co., Ltd. (Jiangsu, China). The mice were housed in polypropylene cages and kept in a room at 25°C and 50% relative humidity under a 12 h light/dark cycle.

The mice were adaptively fed with a normal diet for 1 week. Based on our previous research (Wu et al., 2017; Xu et al., 2021), ApoE^−/−^ mice were fed with a high-fat diet (HFD, containing 21% fat and 0.15% cholesterol) to replicate the atherosclerotic disease model. After 10 weeks of HFD, all the ApoE^−/−^ mice were divided into five groups (n = 10): model group, Gualou–Xiebai group (6 g/kg), Gualou group (4 g/kg), Xiebai group (2 g/kg), and atorvastatin group (10 mg/kg). In a word, all the drugs were administrated to AS mice via oral gavage for 4 weeks. In addition, C57BL/6 mice were fed a normal diet throughout the experiment as the control group. At the end of the experiment, mice were sacrificed and the serum and aorta of mice were collected for further investigation.

### 2.5 Pathophysiological observation

Whole arteries, including the aortic arch, thoracic, and abdominal regions, were cut longitudinally, fixed, and then stained with oil-red O (ORO) staining for lipid measurement at the surface of the vascular wall. Photographs were captured using a high-resolution camera.

In addition, aortic sinus and arch specimens embedded in paraffin were sectioned at a thickness of 4 μm, and slides were stained with hematoxylin and eosin (HE) or collagen-specific Masson. Slides were observed with the microscope (Carl Zeiss, China) and digitally photographed. The proportion of plaque area was calculated using Image-Pro Plus 6.0 (Media Cybernetics, United States).

### 2.6 HPLC-Q-TOF-MS analysis

The herbs extracts were subjected to chromatographic analysis on a Waters Acquity™ HPLC system (Waters, United States) equipped with a Topsil™ C_18_ column (4.6 × 250 mm, 5 μm) and the Waters Xevo G2 Q-TOF mass spectrometer (Waters, United States) equipped with an ESI source.

The chromatographic conditions were as follows. The mobile phase consisted of 0.05% formic acid in water (A) and 0.05% formic acid in acetonitrile (B). The temperature of the column oven was maintained at 35°C. The separation was effected using a linear gradient with a flow of 0.3 ml/min as follows (time/B%): 0–2.5 min, 5% B; 2.5–5 min, 5%–6% B; 5–10 min, 6%–12% B; 10–40 min, 12%–69% B; 40–45 min, 69%–100% B; 45–47 min, 100%–5% B; 47–57 min, 5% B.

The mass spectrometer conditions were as follows. The acquisition was carried out in negative and positive mode, and the mass range was 50–1,200 Da, with the capillary voltage of 2.5 and 3.0 kV, sampling cone voltage of 50 and 40 V respectively, and source temperature of 110°C (ESI^−^) or 120°C (ESI^+^), respectively. The extraction cone voltage was 4.0 V, using cone gas flow of 50 L/h, desolvation gas (N_2_) flow of 600 L/h, and desolvation gas temperature of 350°C. The collision voltage of low and high energy scans is respectively set to 6.0 and 20–80 eV. The accurate mass and composition of the unknown compound ions can be calculated via MassLynx™ V4.1 software (Waters, United States).

The systematic information on GL and XB was collected using the Traditional Chinese Medicine System Pharmacology Database and Analysis Platform (TCMSP) database and literature. According to the imported table information required using UNIFI software, a self-building library including compound name, molecular formula, chemical structure, and accurate molecular mass was constructed. Mass spectrometry data and library were imported into the software for analysis with the unknown compound ions combined with positive adducts including H^+^, Na^+^, and negative adducts containing HCOO^−^ and H^+^.

### 2.7 Network pharmacology analysis

#### 2.7.1 Compounds screening

The compounds of GLXB extract were detected using HPLC-Q-TOF-MS and supplemented via metabolite profiling of plasma and urine of GLXB which had been reported (Lin et al., 2018). According to the imported table information required by UNIFI software, the systematic information of GLXB extract was constructed.

#### 2.7.2 Targets prediction of Gualou–Xiebai against atherosclerosis

The targets of the chemical compound of GLXB in AS treatment were identified by following steps. First, the corresponding targets of the compounds of GLXB were collected via the Swiss Target Prediction (http://swisstarget prediction. ch/), similarity ensemble approach (SEA, http://sea.bkslab.org/), and TCMSP (http://tcmspw.com/). Second, the targets of AS were predicted using TTD (http://bidd.nus.edu.sg/group/cjttd/), OMIM (http://www.omim.org/), DrugBank (https://www.genecards.org/), and Genecards (https://www.genecards.org/) database. It is worth noting that only the targets of Homo sapiens filtered using UniProt (https://www.uniprot.org/) were further analyzed. At last, the two subsets of the target obtained from the above steps were cross-referenced using the Venn diagram package.

#### 2.7.3 Construction of the compound–target network

The interaction information of targets was gained using the STRING (https://string-db.org/) database. The chemical components of GLXB and its therapeutic targets in AS were constructed using Cytoscape 3.6.1 (https://cytoscape.org/) to construct the compound–target network. Topological parameters of the network (degree, betweenness centrality, and closeness centrality) were analyzed using the tools Network Analyzer of Cytoscape 3.6.1. The major active compounds and therapeutic targets were screened by the value of topological parameters higher than the median.

#### 2.7.4 Gene function annotation and construction of the target-pathway/biological process network

The Database for Annotation, Visualization, and Integrated Discovery (DAVID) (Version 6.8) (https://david.ncifcrf.gov/) provided systematic and comprehensive biological function annotation information for a large number of genes. We introduced the target set of GLXB for AS treatment into DAVID and defined the species as “Homo sapiens” for Gene Ontology (GO) and Kyoto Encyclopedia of Genes and Genomes (KEGG) pathway analyses. GO enrichment analysis included biological processes, molecular functions, and cellular components, which annotated the biological functions of genes and discovered the molecular mechanisms of GLXB in treating AS. A *p* value < 0.01 was used as a screening condition. Enrichment analysis bubble maps were plotted using the R language. Based on the results of the KEGG pathway analysis, pathways related to GLXB and the top 20 enriched genes were identified. Then, Cytoscape (version 3.6.1) was used to further construct the target-pathway/biological process network.

### 2.8 Experimental validation

#### 2.8.1 Biochemical analysis

Serum levels of TC, TG, LDL-C, and HDL-C were analyzed via an enzyme-labeled instrument (Molecular Devices Co., Ltd. California, United States). The serum levels of IL-1β, IL-6, TNF-α, ALOX5, PTGS2, VEGFA, and eNOS were determined using ELISA kits following the manufacturer’s instructions.

#### 2.8.2 Western blot analysis

The total proteins from the aortic tissue were extracted. Then, protein samples were separated using sodium dodecyl sulfate–polyacrylamide gel electrophoresis (SDS-PAGE) and transferred onto the polyvinylidene fluoride (PVDF) membrane. After being blocked in 5% skimmed milk for 2 h, the membranes were incubated at 4°C overnight with the primary antibodies, including β-actin, VCAM-1, ICAM-1, GAPDH, p38, p-p38, ATK1, and p-ATK1. Then, the membranes were incubated with corresponding horseradish peroxidase-conjugated secondary antibodies.

### 2.9 Coimmunofluorescence staining

Frozen sections of aortic tissue were prepared and rewarmed to room temperature before the experiment. The frozen sections were fixed with 4% formaldehyde for 30 min and permeabilized with 0.1% Triton X-100 in PBS for 40 min. Then the goat serum was added and sealed at 37°C for 60 min. After incubating with primary antibodies at 4°C overnight, the slices were washed with PBS before incubation with appropriate fluorescence-labeled secondary antibodies at 37°C for 1 hour. Then, the slices were added DAPI at room temperature for 10 min before taking photographs under a laser microscope.

### 2.10 Statistical analysis

At least three independent replications were performed. All data were presented as mean ± SEM and analyzed using SPSS 23.0 (United States). Images were processed using Graphpad Prism 5 (GraphPad Software, United States) and Adobe Photoshop (Adobe, United States). Before the statistical analysis, the data were detected to obey normal distribution. Two-tailed Student’s t test was used for analysis between two groups. One-way ANOVA followed by Tukey post hoc tests was used for analysis when more than two treatments were compared. *p* < 0.05 was considered statistically significant.

## 3 Results

### 3.1 The synergetic anti-atherosclerotic effects of Gualou–Xiebai herb pair

**FIGURE 1:**
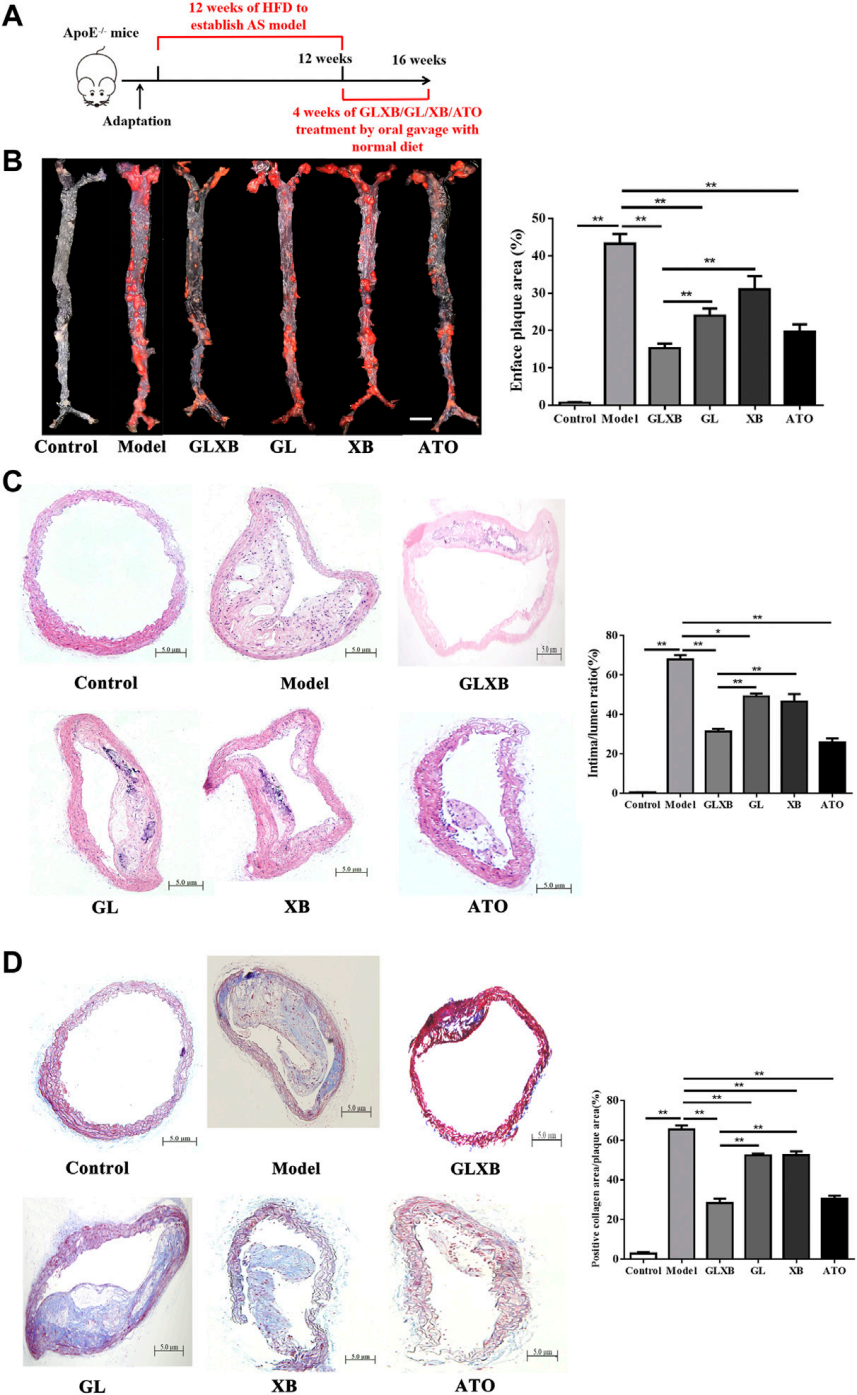
The synergetic anti-atherosclerotic effects of GLXB herb pair. **(A)** Study design of *in vivo* experiment. **(B)** *En face* oil-red O (ORO) staining on the whole aorta of mice and quantification of plaque areas. Bar = 3 mm. (n = 3). **(C)** HE staining on aortic arch and quantification of plaque areas. Bar = 500 μm. (n = 6). **(D)** Masson staining on aortic arch and quantification of plaque areas. Bar = 500 μm. (n = 6). Data were expressed as mean ± SEM. ^*^ *p* < 0.05, ^**^ *p *< 0.01. GLXB: Gualou–Xiebai herb pair (6 g/kg); GL: Gualou (4 g/kg); XB: Xiebai (2 g/kg); ATO: Atorvastatin (10 mg/kg).

To evaluate the synergistic anti-atherosclerotic effects of the GLXB herb pair, we detected plaque formation in the aorta of mice. AS mice were established by feeding with HFD for 12 weeks, and the examination groups were given intragastric administration daily for a total of 4 weeks based on our previous study (Figure 1A). The results of ORO staining and HE staining revealed that the aortas of mice were severely covered by atherosclerotic plaques after HFD administration (*p *< 0.01), whereas GLXB treatment (6 g/kg) inhibited atherosclerotic lesion formation compared with the model group (*p *< 0.01) (Figures 1B,C). In addition, Masson staining showed that GLXB treatment (6 g/kg) significantly reduced the collagen deposition in the aortic vessels (*p *< 0.01) (Figure 1D). Furthermore, the anti-atherosclerotic effects of the GLXB herb pair (6 g/kg) were more significant than those of Gualou (4 g/kg) and Xiebai (2 g/kg) (*p *< 0.01). Atorvastatin (10 mg/kg) could significantly inhibit plaque formation and collagen deposition, indicating the feasibility of this experiment (*p *< 0.01). These results confirmed that GLXB could reduce atherosclerotic plaques in AS mice, and GLXB herb pair treatment was more effective than Gualou or Xiebai alone.

### 3.2 Identification of the main components from Gualou–Xiebai using HPLC-Q-TOF-MS

**FIGURE 2:**
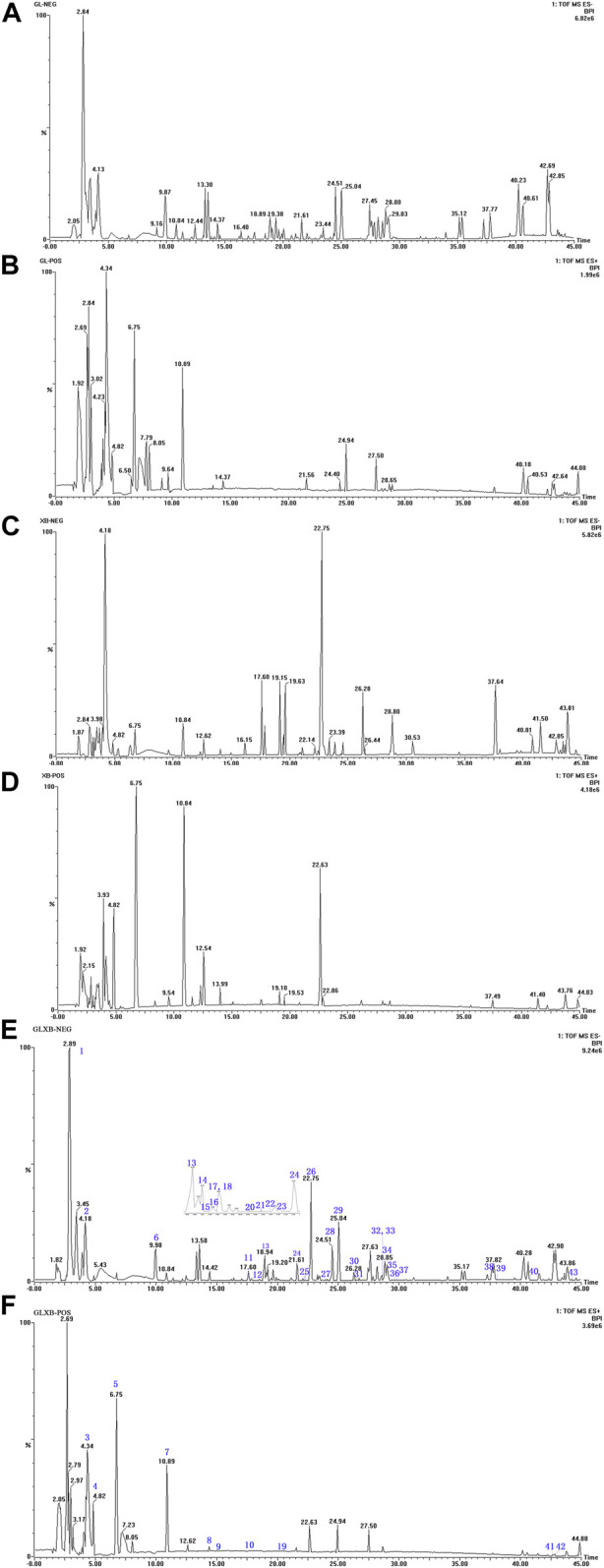
The base peak intensity (BPI) chromatograms of GLXB and single herbs from HPLC-Q-TOF-MS analysis. **(A)** Negative scan of Gualou; **(B)** positive scan of Gualou; **(C)** negative scan of Xiebai; **(D)** positive scan of Xiebai; **(E)** negative scan of Gualou–Xiebai; **(F)** positive scan of Gualou–Xiebai.

To figure out why the GLXB herb pair was more effective than Gualou or Xiebai alone, we first identified the main components from GLXB and the single herbs using HPLC-Q-TOF-MS. The quality assessment of GLXB based on HPLC fingerprints was shown in the supplementary material. The base peak intensity (BPI) chromatograms of GLXB and the single herbs in positive and negative ion modes were depicted in Figure 2.

Then, the UNIFI screening platform was utilized for the analysis of MS data. After automatic information matching and further manual verification, 43 common compounds were ultimately identified from the GLXB herb pair, including 17 steroid saponins, eight flavonoids, seven organic acids, five tetracyclic triterpenoids, two amino acids, two nucleosides, and two other compounds. The detailed MS information of these components was summarized in Table 1. Furthermore, in order to supplement the metabolite prototype of herb pair *in vivo*, we added the following five prototypes of metabolites deriving from GLXB herb pair in plasma and urine of rats based on the reported study (Li et al., 2019), including macrostemonoside B, 25S-macrostemonoside B, apigenin, chrysoeriol, and kaempferol. Therefore, 48 main components from the GLXB herb pair were identified as active components.

**TABLE 1:** Analysis of compounds of GLXB using HPLC-Q-TOF-MS^E^.

No.	RT (min)	PubChem CID	Chemical name	Chemical formula	Neutral mass (Da)	Observed neutral mass (Da)	Experimental mass (m/z)	Error ppm	MS and MS^E^ data (+ or −) (m/z)
1	2.89	444,173	Beta-D-arabinopyranose	C_5_H_10_O_5_	150.0528	150.0505	195.0494	5.64	195.0494[M + HCOO]^−^
2	4.18	16,590	Diallyl disulfide	C_6_H_10_S_2_	146.02239	146.0216	191.0198	4.2	191.0198[M + HCOO] ^−^
3	4.34	60,961	Adenosine	C_9_H_13_O_4_N_5_	267.09675	267.0976	268.19	2.5	268.10497[M + H]^+^
									136.06274 [M + H − Rib]^+^
4	4.82	135398635	Guanosine	C_5_H_5_N_5_O	283.0917	283.0911	284.0983	−1.2	284.0983[M + H]^+^
									152.0565[M + H − Rib]^+^
5	6.76	6,140	L-phenylalanine	C_9_H_11_NO_2_	165.0793	165.0788	166.0861	−0.8	166.0861[M + H]^+^
6	9.98	14132336	Vanillic acid 4-β-D-glucoside	C_14_H_18_O_9_	330.0884	330.29	329.0857	8.204	659.1829[2M − H]^−^
									329.0857[M − H] ^−^
7	10.89	1,148	DL-Tryptophan	C_11_H_12_N_2_O_2_	204.0899	204.0899	205.0972	0.1	205.0972[M + H]^+^
8	14.37	98,285	2,3,4,9-tetrahydro-1H-pyrido [3,4-b] indole-3-carboxylic acid	C_12_H_12_N_2_O_2_	216.0899	216.0897	217.0970	−0.9	217.0970[M + H]+
									144.0807[M + H − C2H3NO2]+
9	15.10	148,385	(1S,3S)-2,3,4,9-Tetrahydro-1-methyl-1H-pyrido[3,4-B]indole-3-carboxylic acid	C_13_H_14_N_2_O_2_	230.10553	230.1063	231.1136	−4.76	231.1136[M + H]^+^
									158.0987[M + H − C2H3NO2]^+^
									143.5335[M + H − C3H6NO2]^+^
10	17.55	131751143	Chinenoside V	C_45_H_72_O_19_	916.4741	916.4741	917.4784	−4.69	917.4784[M + H]^+^
11	17.60	-	26-[(β-D-glucopyranosyl)oxy]-2β,22-dihydroxy-5β-furostan-25(27)-en-3β-yl-O-β-D-glucopyranosyl-(1→2)-β-D-galactopyranoside	C_45_H_72_O_20_	934.4773	934.4749	977.4770	2.568	979.4770[M + HCOO]^−^
									933.47046[M − H] ^−^
									771.4152[M – H − Glc] ^−^
12	17.85	101669618	Macrostemonoside J	C_45_H_76_O_20_	936.4930	936.4932	981.4914	−2.95	981.4914[M + HCOO] ^−^
13	18.94	5280805	Rutin	C_27_H_30_O_16_	610.15338	610.1532	609.1459	3.94	609.1459[M − H] ^−^
14	19.20	101923513	Macrostemonoside I	C_45_H_72_O_20_	932.4617	932.4564	931.4564	0.614	977.4615[M + HCOO] ^−^
									931.4564[M − H] ^−^
15	19.38	5318767	Kaempferol-3-O-β-rutinoside	C_27_H_30_O_15_	594.15847	594.1577	593.1505	−1.2	593.1507[M − H] ^−^
16	19.48	101923511	Macrostemonoside G	C_45_H_74_O_20_	934.47734	934.4781	979.4763	−0.1	979.4763[M + HCOO] ^−^
									934.4781[M − H] ^−^
17	19.61	5280459	Quercitrin	C_21_H_20_O_11_	448.1006	448.1002	447.0928	−1.1	447.0928[M − H] ^−^
									285.03907[M – H − OGlc] ^−^
18	19.63	-	Macrostemonoside P	C_45_H_76_O_20_	936.49299	936.4927	981.4909	−2.4	981.4909[M + HCOO] ^−^
									935.4830[M − H] ^−^
19	20.39	5280343	Quercetin	C_15_H_10_O_7_	302.0427	302.0427	303.0505	9.569	303.0505[M + H]^+^
20	20.44	-	Quercetin-3-O-α-D-riboside	C_20_H_18_O_11_	434.0849	434.0779	433.0776	−0.69	433.0776[M − H] ^−^
21	20.75	-	Kaempferol-3-D-glucoside-7-O-rhamnoside	C_27_H_30_O_14_	578.1636	578.1634	577.1616	0.1	577.1564[M − H] ^−^
22	20.98	-	25R-Macrostemonoside P	C_45_H_76_O_20_	936.4930	936.4932	981.4914	−2.95	981.4914[M + HCOO] ^−^
									935.4930[M − H] ^−^
									773.4301 [M – H − Glc] ^−^
23	21.23	5385553	Apigenin-7-O-β-D-glucoside	C_21_H_20_O_10_	432.1056	432.0984	431.0984	−3.48	431.0984[M − H] ^−^
24	21.61	-	Chrysoeriol-7-O-β-D-glucoside	C_22_H_22_O_11_	462.1162	462.1154	461.1081	0	461.1081[M − H] ^−^
									283.02409[M – H − C_6_H_10_O_6_] ^−^
25	22.47	-	Macrostemonoside O	C_45_H_74_O_19_	918.48243	918.4821	963.4803	−0.3	963.4803[M + HCOO] ^−^
									918.4821[M − H] ^−^
26	22.75	123134749	Timosaponin BII	C_13_H_14_N_2_O_2_	920.49808	920.4994	965.4995	0.10	965.4995[M + HCOO] ^−^
									919.4907[M − H] ^−^
27	23.92	137701652	25R-timosaponin BII	C_13_H_14_N_2_O_2_	920.49808	920.4994	965.4995	−1.52	965.4995[M + HCOO] ^−^
									919.4907[M − H] ^−^
28	24.51	44406496	Bryoamaride	C_36_H_54_O_12_	678.3583	678.3602	723.3584	−2.8	723.3596[M + HCOO] ^−^
									677.3538[M − H]^−^
29	25.04	101306926	25-O-Acetylbryoamaride	C_38_H_56_O_13_	720.3721	720.3729	765.3712	1.1	765.3712[M + HCOO]^−^
									719.3661[M − H]^−^
30	26.28	192,523	Macrostemonoside F	C_45_H_74_O_18_	902.48752	902.4882	947.4864	0.7	947.4864[M + HCOO]^−^
									901.4882[M − H]^−^
31	26.44	101389834	Timosaponin C	C_45_H_74_O_18_	902.4875	902.4882	947.4877	2.551	947.4864[M + HCOO]^−^
									901.4882[M − H]^−^
32	28.60	5281318	Cucurbitacin D	C_30_H_44_O_7_	516.3087	516.3087	516.3092	1.553	561.3074[M + HCOO]^−^
33	28.60	-	Macrostemonoside S	C_39_H_62_O_14_	754.4145	754.4067	799.4114	0.133	799.4114[M + HCOO]^−^
									753.4122[M − H]^−^
34	28.85	5321949	Tianshic acid	C_18_H_34_O_5_	330.2406	330.2442	329.2406	−2.73	329.2406[M − H]^−^
35	29.03	5281316	Cucurbitacin B	C_32_H_46_O_8_	558.3193	558.3196	603.3178	0.6	603.3178[M + HCOO]^−^
									557.3191[M − H]^−^
36	29.51	5352014	Isocucurbitacin B	C_32_H_46_O_8_	558.3193	558.3098	603.3191	−3.65	603.3191[M + HCOO]^−^
									557.3098[M − H]^−^
37	29.76	13,849	Pentadecanoic acid	C_15_H_30_O_2_	242.2246	242.2212	287.2222	3.482	287.22222[M + HCOO]^−^
38	37.68	71306914	Timosaponin A III	C_39_H_64_O_13_	740.4371	740.4347	785.4356	0.8	785.4335[M + HCOO]^–^
									739.4353[M − H]^−^
									577.3734[M − H − Glc]^−^
39	38.06	197,480	Macrostemonoside A	C_51_H_84_O_23_	1064.5403	1064.5375	1109.5357	2.6	1109.53566[M + HCOO]^−^
40	40.87	197,481	Macrostemonoside D	C_53_H_86_O_24_	1106.5509	1106.5509	1151.5491	0	1151.5491[M + HCOO]^−^
									1105.5338[M − H]^−^
41	42.64	5281126	Punicic acid	C_18_H_30_O_2_	278.2246	278.2324	279.2333	−3.22	279.2333[M + H]^+^
42	42.80	5280934	Linolenic acid	C_18_H_30_O_2_	278.2246	278.2241	279.2313	−3.22	279.2313[M + H]^+^
43	44.19	5742590	Sitogluside	C_35_H_60_O_6_	576.4390	576.4389	621.4371	−0.1	621.4374[M + HCOO]^−^

**FIGURE 3:**
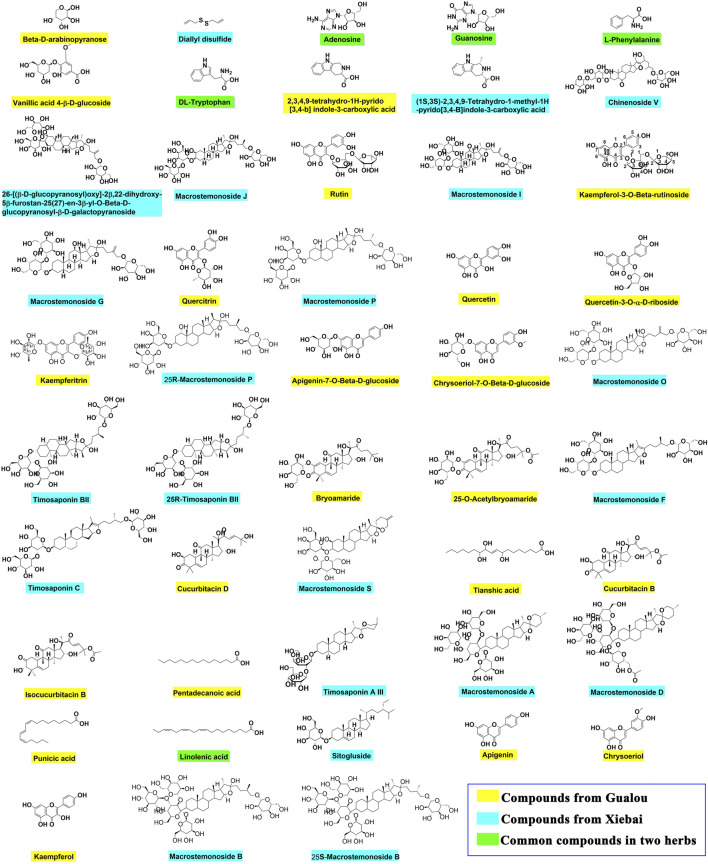
The chemical structures of main compounds from GLXB and the single herbs based on analysis of HPLC-Q-TOF-MS and information retrieval. Compounds from Gualou: labeled as yellow nodes; compounds from Xiebai: labeled as blue nodes; common compounds in two herbs: labeled as green nodes.

Among the identified 48 compounds from the GLXB herb pair, 27 compounds were from Gualou (labeled as yellow) and 26 compounds were from Xiebai (labeled as blue), and the two herbs shared five compounds (labeled as green), according to the component identification of the single herbs (Figure 3). In addition, flavonoids, tetracyclic triterpenoids, and organic acids were the main components of Gualou, whereas steroidal saponins were the main components of Xiebai. These results indicated that the quantity of components from the GLXB herb pair was much more than Gualou or Xiebai alone.

### 3.3 Target prediction and functional analysis of Gualou–Xiebai on atherosclerosis using network pharmacology

#### 3.3.1 Target prediction of Gualou–Xiebai on atherosclerosis

**FIGURE 4:**
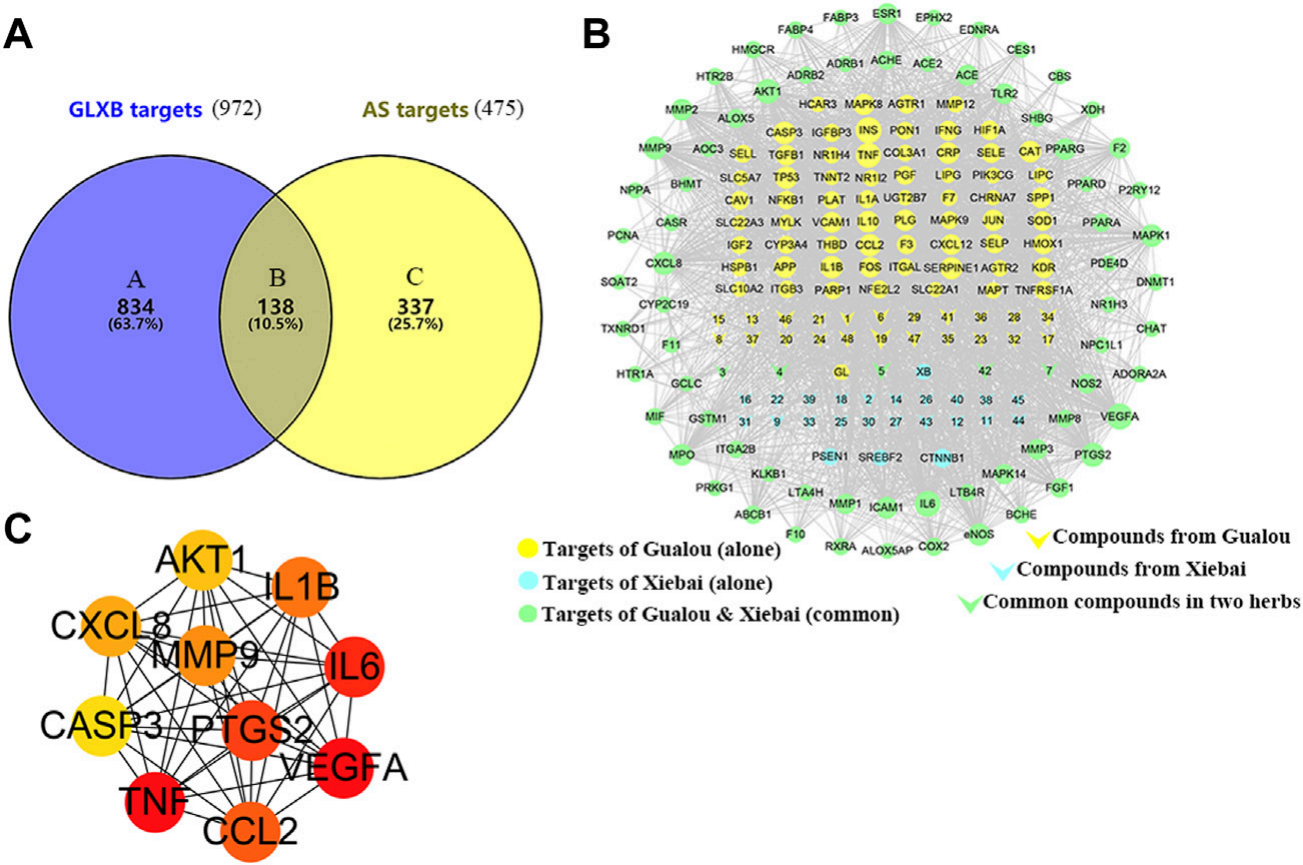
Target maps of GLXB on AS. **(A)** The Venn diagram of the target of GLXB against AS. **(B)** Compound–target network of GLXB. Targets of Gualou: labeled as yellow circle nodes; targets of Xiebai: labeled as blue circle nodes; targets of Gualou and Xiebai: labeled as green circle nodes; compounds from Gualou: labeled as yellow triangle nodes; compounds from Xiebai: labeled as blue triangle nodes; common compounds in two herbs: labeled as green triangle nodes. **(C)** Ten key targets of GLXB on AS.

To determine the target of GLXB for treating AS, Swiss Target Prediction, SEA, and TCMSP were introduced to predict the targets of the active compounds. At last, a total of 138 AS-related targets regulated using 48 active compounds were determined, which were ultimately identified as target genes of GLXB for the treatment of AS (Figure 4A). Furthermore, to explore the combined mechanisms of GLXB for AS treatment, 48 active compounds and 138 targets were used to construct the compound–target network. There were 135 targets regulated using 27 active compounds from Gualou (labeled as yellow) and 73 targets regulated using 26 active compounds from Xiebai (labeled as blue), and the two herbs shared 70 targets (labeled as green) (Figure 4B).

Furthermore, 21 major compounds with 56 anti-atherosclerotic targets of GLXB were screened based on the criterion of the topological eigenvalue (Table 2). Then, 10 key targets were screened via the MCC algorithm, listing as VEGFA (Vascular endothelial growth factor A), TNF (tumor necrosis factor), IL6 (Interleukin 6), PTGS2 (Prostaglandin-endoperoxide synthase 2), CCL2 (C-C motif chemokine 2), IL1B (Interleukin 1B), MMP9 (Matrix Metallopeptidase 9), CXCL8 (Interleukin 8), AKT1 (Serine/threonine-protein kinase), CASP3 (Caspase 3) (Figure 4C). These results indicated that the GLXB herb pair handled more anti-atherosclerotic targets than Gualou or Xiebai alone.

**TABLE 2:** Topological eigenvalues of the major compounds and targets.

	NO.	Name	Degree	Betweenness centrality	Closeness centrality
**Compounds**	1	Quercetin	60	0.01169446	0.57943925
	2	Apigenin	42	0.0064552	0.55029586
	3	PHA	38	0.02387083	0.55029586
	4	Guanosine	34	0.01194087	0.53448276
	5	Tianshic acid	33	0.01372401	0.50819672
	6	Linolenic acid	33	0.013192	0.53757225
	7	Chrysoeriol	28	0.00833858	0.52394366
	8	Pentadecanoic acid	27	0.01166515	0.50543478
	9	Punicic acid	27	0.00488589	0.49206349
	10	Kaempferol	25	0.00401259	0.51955307
	11	WV	22	0.00470239	0.5027027
	12	Vanillic acid 4-β-D-glucopyranoside	17	0.00603805	0.496
	13	Rutin	15	0.00054976	0.48186528
	14	Apigenin-7-O-β-D-glucoside	14	0.00343475	0.49076517
	15	Chrysoeriol-7-O-β-D-glucoside	13	0.00172374	0.48691099
	16	Cucurbitacin D	13	0.00125008	0.45588235
	17	Sitogluside	13	0.00302562	0.46969697
	18	Quercitrin	11	0.00061121	0.45925926
	19	Quercetin-3-O-α-D-riboside	10	0.00106769	0.47328244
	20	2,3,4,9-tetrahydro-1H-pyrido [3,4-b] indole-3-carboxylic acid	8	0.00052686	0.4503632
	21	Adenosine	7	0.00132463	0.4503632
**Targets**	1	VEGFA	103	0.118007	0.681319
	2	INS	102	0.053024	0.678832
	3	IL6	101	0.036807	0.686347
	4	TNF	93	0.032016	0.661922
	5	AKT1	92	0.025733	0.657244
	6	PTGS2	85	0.026923	0.632653
	7	CXCL8	79	0.015883	0.626263
	8	MMP9	76	0.009823	0.61794
	9	MAPK1	73	0.020067	0.611842
	10	CASP3	72	0.009116	0.605863
	11	IL1B	71	0.008164	0.598071
	12	TP53	71	0.014121	0.605863
	13	eNOS	71	0.021831	0.603896
	14	CCL2	69	0.006417	0.601942
	15	SERPINE1	68	0.009258	0.594249
	16	ICAM1	66	0.010595	0.592357
	17	MMP2	64	0.004805	0.586751
	18	MPO	64	0.008849	0.590476
	19	IL10	63	0.004539	0.58125
	20	PPARG	63	0.009372	0.588608
	21	CAT	63	0.012122	0.583072
	22	APP	63	0.024471	0.58125
	23	CRP	61	0.006157	0.575851
	24	MAPK8	60	0.00444	0.574074
	25	PLG	60	0.007493	0.583072
	26		60	0.012099	0.58125
	27	JUN	59	0.004203	0.575851
	28	ESR1	59	0.012214	0.579439
	29	F2	58	0.015548	0.57764
	30	VCAM1	57	0.002853	0.56535
	31	TLR2	56	0.005041	0.575851
	32	FOS	55	0.004311	0.56535
	33	TGFB1	54	0.002668	0.56535
	34	KDR	54	0.010205	0.574074
	35	CXCL12	54	0.007289	0.574074
	36	MMP3	53	0.004459	0.563636
	37	SPP1	53	0.003758	0.567073
	38	HMOX1	52	0.00349	0.561934
	39	p38	52	0.005175	0.567073
	40	MMP1	51	0.003625	0.555224
	41	NOS2	46	0.006216	0.548673
	42	HIF1A	44	0.007308	0.542274
	43	FGF1	44	0.047875	0.542274
	44	SOD1	43	0.006697	0.540698
	45	SELP	42	0.003888	0.551929
	46	F3	41	0.004114	0.537572
	47	ALOX5	41	0.007343	0.540698
	48	ACHE	40	0.013301	0.529915
	49	CTNNB1	39	0.003229	0.53913
	50	AGTR1	37	0.002639	0.536023
	51	PGF	36	0.003545	0.528409
	52	PPARA	36	0.009191	0.518106
	53	ABCB1	36	0.019244	0.532951
	54	PTGS1	33	0.004986	0.531429
	55	CYP3A4	30	0.009208	0.522472
	56	MMP8	29	0.002706	0.519553

#### 3.3.2 Functional enrichment and pathways analysis of Gualou–Xiebai on atherosclerosis

**FIGURE 5:**
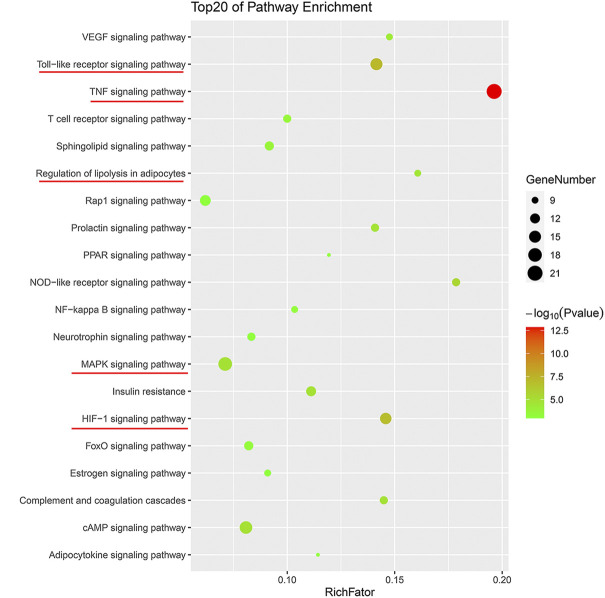
Top 20 enriched pathways of GLXB on AS.

**FIGURE 6:**
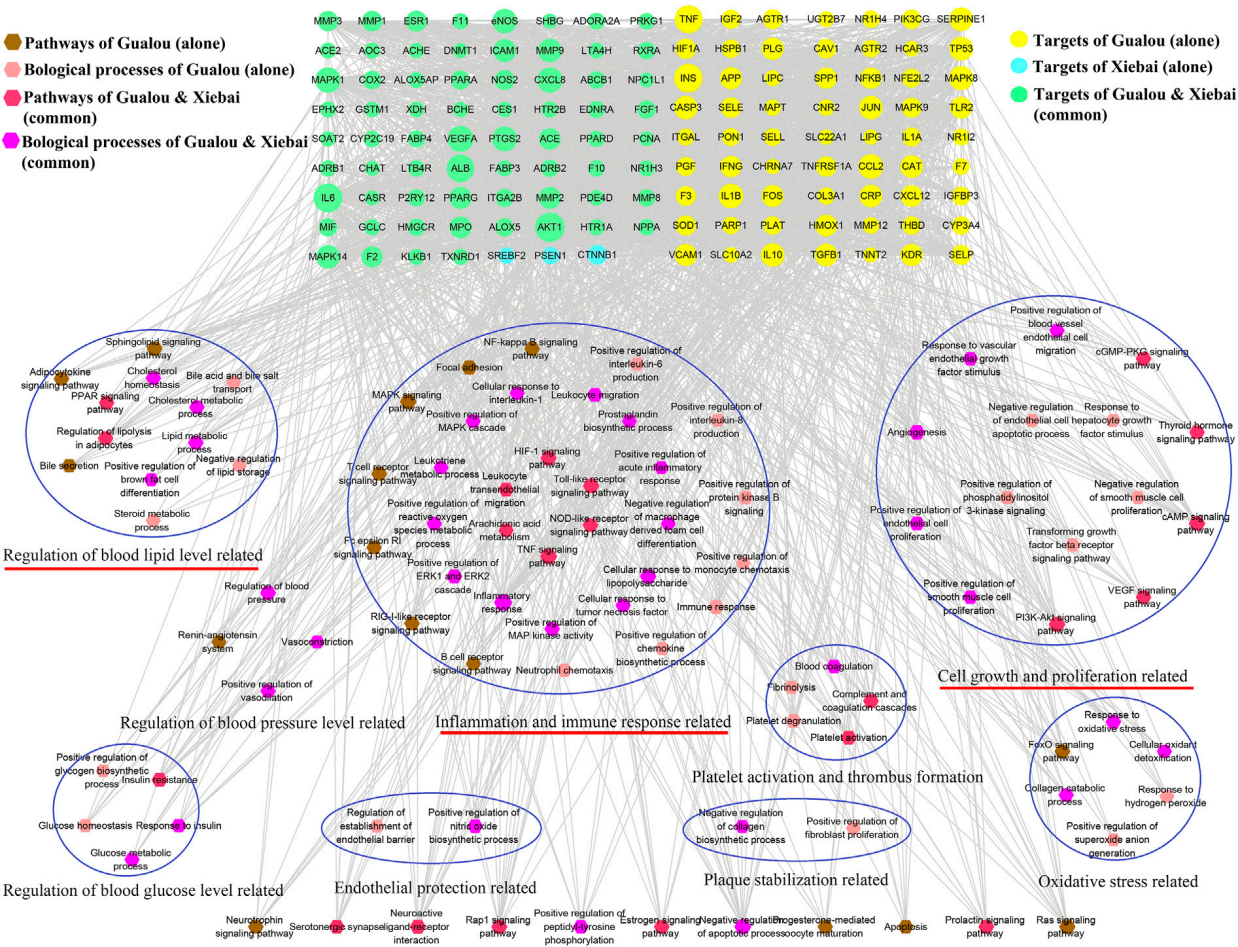
Target-pathway/biological process network of GLXB. Pathways of Gualou: labeled as brown hexagon nodes; biological process of Gualou: labeled as pink hexagon nodes; pathways of Xiebai: labeled as red hexagon nodes; biological process of Xiebai: labeled as purple hexagon nodes; targets of Gualou: labeled as yellow circle nodes; targets of Xiebai: labeled as blue circle nodes; targets of Gualou and Xiebai: labeled as green circle nodes.

To further clarify the combination pathways of GLXB for treating AS, all 138 potential targets of GLXB were mapped onto KEGG pathways with *p* < 0.05. In addition, the top 20 enriched pathways were selected based on the threshold of FDR <0.01 (Figure 5). Furthermore, a compound–target–pathway network was constructed, containing 219 nodes with 48 representative components, 138 representative targets, 37 representative pathways, 58 biological processes, and 2,888 edges. Gualou and Xiebai shared 21 signaling pathways (labeled as red) and 35 biological processes (labeled as purple), whereas 16 signaling pathways (labeled as brown) and 23 biological processes (labeled as pink) were regulated by Gualou alone (Figure 6). These results confirmed that the GLXB herb pair regulated more signaling pathways and more biological processes for treating AS than Gualou or Xiebai alone.

Among the 37 pathways, the TNF signaling pathway exhibited a relatively high number of target connections (degree = 21), followed by the MAPK signaling pathway (degree = 18), toll-like receptor signaling pathway (degree = 15), and HIF-1 signaling pathway (degree = 14), etc., all of which were related to inflammation and immune response. In addition, regulation of lipolysis in adipocytes (degree = 9) and VEGF signaling pathway (degree = 9) also had a high degree, which were related to the regulation of blood lipid level and endothelial growth/function.

Therefore, according to the compound–target–pathway network, the possible mechanisms of GLXB treatment against AS mainly included three aspects, inflammation/immune response, endothelial growth/function, and regulation of blood lipid levels.

### 3.4 Validation of the mechanisms of Gualou–Xiebai treatment against atherosclerosis *in vivo*

#### 3.4.1 Gualou–Xiebai suppressed inflammatory response of atherosclerosis mice

**FIGURE 7:**
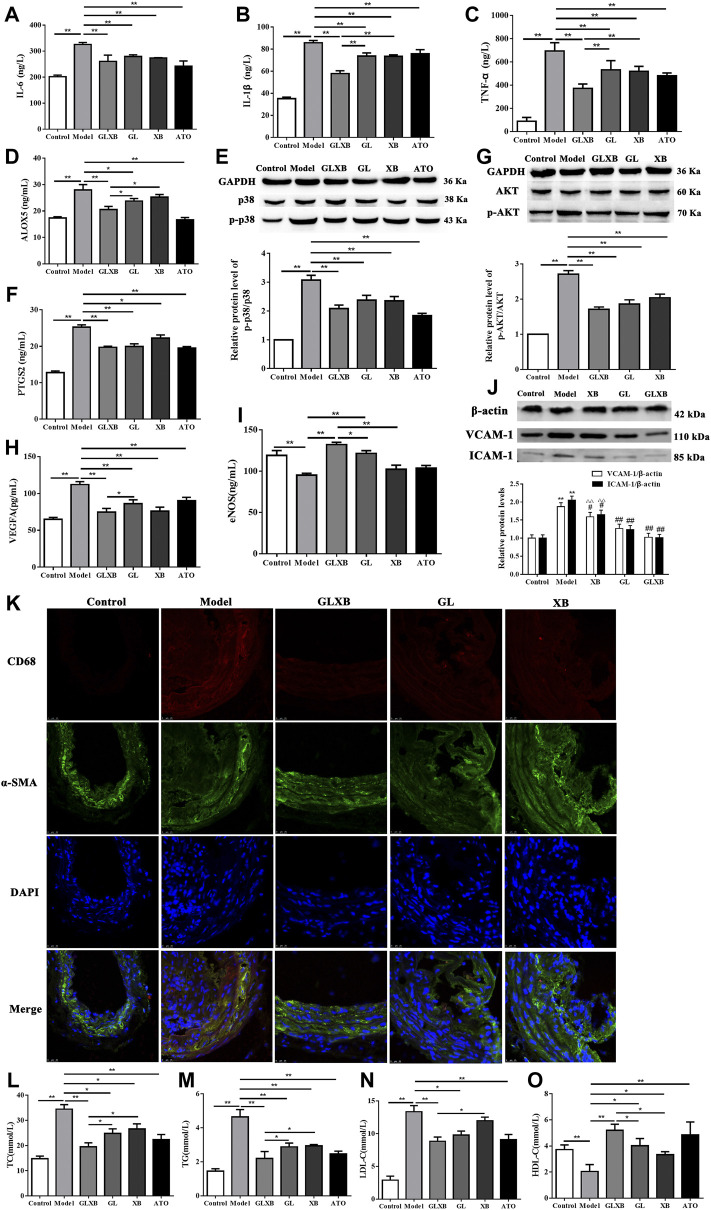
Validation of the mechanisms of GLXB treatment against AS *in vivo.* **(A)** The level of IL-6 in the serum of mice. **(B)** The level of IL-1β in the serum of mice. **(C)** The level of TNF-α in the serum of mice. **(D)** The level of ALOX5 in the serum of mice. **(E)** The expression of the p-p38 protein in the aorta of mice. **(F)** The level of PTGS2 in the serum of mice. **(G)** The expression of the p-AKT protein in the aorta of mice. **(H)** The level of VEGFA in the serum of mice. **(I)** The level of eNOS in the serum of mice. Data were expressed as mean ± SEM. ^*^ *p* < 0.05, ^**^ *p* < 0.01. **(J)** The expressions of VCAM-1 and ICAM-1 proteins in the aorta of mice. Data were expressed as mean ± SEM. ^**^ *p* < 0.01 vs. control group, ^ΔΔ^ *p *< 0.01 vs. GLXB group, ^#^ *p* < 0.05, ^*##*^ *p *< 0.01 vs. model group. **(K)** Coimmunofluorescence staining of aortic tissue sections with antibodies to CD68 and α-SMA. Bar = 25 μm. **(L)** The level of TC in the serum of mice. **(M)** The level of TG in the serum of mice. **(N)** The level of LDL-C in the serum of mice. **(O)** The level of HDL-C in the serum of mice. Data were expressed as mean ± SEM (n = 6). ^*^ *p *< 0.05, ^**^ *p* < 0.01. GLXB: Gualou–Xiebai herb pair (6 g/kg); GL: Gualou (4 g/kg); XB: Xiebai (2 g/kg); ATO: atorvastatin (10 mg/kg).

To clarify the effects of GLXB treatment on the inflammatory response, we selected inflammatory targets screened by network pharmacology for validation. The levels of IL-6, IL-1β, TNF-α, ALOX5, and PTGS2 in serum and the expression of the p-p38 protein in the aorta of AS mice were detected (Figures 7A–F). From the results, the levels of serum IL-6, IL-1β, TNF-α, ALOX5, and PTGS2 and the protein expression of p-p38 were increased in the model group compared with the control group (*p *< 0.01), which were reversed after GLXB treatment (6 g/kg) (*p *< 0.01). In addition, Gualou alone (4 g/kg) could decrease these targets, whereas Xiebai alone (2 g/kg) could inhibit IL-6, IL-1β, TNF-α, and PTGS2 levels and p-p38 expression, compared with the model group (*p *< 0.05, *p *< 0.01). Moreover, the downregulating effects of the GLXB herb pair (6 g/kg) on the levels of IL-1β, TNF-α, and ALOX5 were more significant than those of Gualou (4 g/kg) and Xiebai alone (2 g/kg) (*p *< 0.05, *p *< 0.01). These results indicated that the GLXB herb pair played an antiinflammatory role in AS mice.

#### 3.4.2 Gualou–Xiebai regulated the vascular endothelial growth/function of atherosclerosis mice

To illustrate the effects of GLXB treatment on endothelial cell growth and endotheliocyte function, we selected endothelial growth-related targets screened by network pharmacology and adhesion indicators for validation. The levels of VEGFA, eNOS in serum, the expressions of p-AKT, VCAM-1, and ICAM-1 proteins in the aorta, and macrophage infiltration in the aorta of AS mice were detected.

As shown in Figures 7G–J, serum VEGFA level and p-AKT, VCAM-1, and ICAM-1 proteins expressions were increased in the model group compared to the control group, whereas serum eNOS level was decreased in the model group (*p *< 0.01). Otherwise, the GLXB herb pair (6 g/kg) and Gualou alone (4 g/kg) could reverse the levels above (*p *< 0.01), whereas Xiebai alone (2 g/kg) could significantly decrease the VEGFA level and p-AKT, VCAM-1, and ICAM-1 expressions (*p *< 0.05, *p *< 0.01). The effects of the GLXB herb pair (6 g/kg) on VEGFA and eNOS levels were more significant than those of Gualou alone (4 g/kg), whereas the effects of the GLXB herb pair (6 g/kg) on eNOS, VCAM-1, and ICAM-1 levels were more remarkable than those of Xiebai alone (2 g/kg) (*p *< 0.05).

In addition, we used immunofluorescence to label CD68 as a specific protein marker of macrophages, and α-SMA as a specific protein marker of smooth muscle cells in the aorta. As shown in the results of co-immunofluorescence staining (Figure 7K), the colocalization of CD68 and α-SMA in the aorta was increased in the model group compared with the control group, which could be reversed using the GLXB herb pair. This result indicated that the GLXB herb pair could reduce the adhesion and infiltration of macrophages to the vascular wall.

These results above elucidated that the GLXB herb pair could regulate endothelial growth-related targets and improve endotheliocyte function to alleviate AS.

#### 3.4.3 Gualou–Xiebai regulated the blood lipid levels of atherosclerosis mice

To observe the regulating effects of GLXB treatment on blood lipid levels, we detected the serum levels of TC, TG, LDL-C, and HDL-C in AS mice **(** Figure 7L–O). Compared with the control group, the serum levels of TC, TG, and LDL-C were significantly enhanced and the serum level of HDL-C was reduced in the model group, which could be reversed using GLXB treatment (6 g/kg) (*p *< 0.01). In addition, Gualou alone (4 g/kg) could regulate these blood lipid levels of AS mice and Xiebai alone (2 g/kg) could improve the levels of TC, TG, and HDL-C (*p *< 0.05, *p *< 0.01). Furthermore, the regulating effects of the GLXB herb pair (6 g/kg) on the blood lipid levels were more significant than those of Xiebai alone (2 g/kg), whereas the regulating effects of the GLXB herb pair (6 g/kg) on TC, TG, and HDL-C levels were more obvious than those of Gualou (4 g/kg) (*p *< 0.05). These results demonstrated that the GLXB herb pair could regulate the blood lipid levels to alleviate AS. Moreover, the specific mechanisms of GLXB herb pair on lipid metabolism are still under study.

Together, the validation experiments above illustrated that the synergetic anti-atherosclerotic effects of GLXB herb pair may be because of suppressing inflammation, regulating vascular endothelial growth/function, and improving blood lipid levels.

## 4 Discussion

The main findings of this study were as follows: First, the anti-atherosclerotic effects of the GLXB herb pair (6 g/kg) were more significant than those of Gualou (4 g/kg) or Xiebai alone (2 g/kg). Second, 48 main active components from the GLXB herb pair were identified, which were responsible for the anti-atherosclerotic effects. Third, the GLXB herb pair handled more anti-atherosclerotic targets and more signaling pathways than Gualou or Xiebai alone, whereas 10 key targets of GLXB were found by topological analysis. Fourth, the combination mechanisms of GLXB treatment against AS were verified *in vivo*, including suppressing inflammation, regulating vascular endothelial growth/function, and improving blood lipid levels, and the effects of GLXB herb pair (6 g/kg) on levels of IL-1β, TNF-α, ALOX5, VEGFA, eNOS, VCAM-1, ICAM-1, and blood lipids were more significant than those of Gualou (4 g/kg) or Xiebai alone (2 g/kg).

In our previous study, we found the anti-atherosclerotic effects of GLXB treatment on HFD-fed ApoE^−/−^ mice (Xu et al., 2021). In this study, we confirmed that the GLXB herb pair could inhibit atherosclerotic lesion formation and collagen deposition in the aortic vessels of AS mice. In clinical application of TCM, GLXB herb pair has been used to extinguish phlegm, promote Qi circulation and disperse stasis for thousands of years, which is related to clinical treatment of cardiovascular disease in modern medicine (Ding et al., 2013; Lin et al., 2021). Pharmacological studies have shown that the GLXB herb pair has cardiovascular protective effects (Ding et al., 2016; Yan et al., 2018). In this study, the anti-atherosclerotic effects of the GLXB herb pair (6 g/kg) were proven to be more significant than those of Gualou (4 g/kg) or Xiebai alone (2 g/kg). Therefore, the combination mechanisms of GLXB treatment against AS were worthy of further investigation.

In this study, HPLC-Q-TOF-MS technology was used to rapidly and comprehensively analyze the chemical components of GLXB, and 43 compounds were identified. In consideration of the lack of GLXB’s metabolite prototype in our result, we supplemented five prototypes of metabolites deriving from GLXB herb pair in plasma and urine of rats based on the reported study (Lin et al., 2018; Li et al., 2019). In addition, our results also showed that the quantity of components from the GLXB herb pair was much more than Gualou or Xiebai alone, which was the foundation of the obvious effects of the GLXB herb pair against AS.

Thereafter, to clarify the relationship between compounds and efficacy of GLXB herb pair, network pharmacology was used to predict the anti-atherosclerotic targets and signaling pathways of GLXB treatment based on the main compounds. From our results, the GLXB herb pair handled more anti-atherosclerotic targets and more signaling pathways than Gualou or Xiebai alone, thereby, GLXB herb pair was more effective in treating AS. In addition, 10 key targets of GLXB were found by the topological analysis, listing as VEGFA, TNF, IL6, PTGS2, CCL2, IL1B, MMP9, CXCL8, AKT1, and CASP3. These 10 targets are closely related to the occurrence and development of AS. Studies have shown that abnormal expression of VEGFA could lead to proliferation and migration of vascular endothelial cells, resulting in plaque vulnerability and breakability, macrophage infiltration, and atherosclerotic lesions (Hu et al., 2018; Braile et al., 2020). In addition, the development of AS is closely related to the activation of inflammatory factors, such as TNF-α (Duan et al., 2021; Kim et al., 2021), IL6 (Tyrrell and Goldstein, 2021), IL1B (Mai and Liao, 2020), and CXCL8 (Hedayati-Moghadam et al., 2021). Furthermore, PTGS2 is a rate-limiting enzyme that produces inflammatory prostaglandins, which can enhance inflammation to promote AS (Papageorgiou et al., 2016; Zhou et al., 2021). The study verified that higher expression of the CCL2 gene led to a higher likelihood of developing AS (Winter et al., 2018). Inflammatory cells could accelerate carotid artery calcification via modulating MMP9 and cause AS (Liang et al., 2021). Modulation of the AKT activity levels in macrophages significantly affected their polarization phenotype in AS mice (Linton et al., 2019). CASP3 deletion could promote necrosis in atherosclerotic plaques of ApoE ^−/−^ mice (Grootaert et al., 2016). Taken together, these 10 targets were proven to be involved in the development of AS, which were predicted as the key anti-atherosclerotic targets of GLXB.

Further pathway enrichment analysis revealed that the most important pathways regulated using GLXB were considered as TNF signaling pathway, MAPK signaling pathway, toll-like receptor signaling pathway, HIF-1 signaling pathway, VEGF signaling pathway, and regulation of lipolysis in adipocytes. As reports have shown, the TNF signaling pathway regulates the expressions of many genes related to vascular inflammation (He et al., 2017) and targets inflammatory molecules (Poznyak et al., 2021). The MAPK signaling pathway is involved in inflammatory signaling and promoting the formation of atherosclerotic lesions (Reustle and Torzewski, 2018). The toll-like receptor can reduce the severity of AS by inhibiting vascular inflammation (Li et al., 2020). HIF1α plays multiple roles in the development of AS, including rendering the cells more inflammatory (Knutson et al., 2021). In addition, the VEGF signaling pathway could lead to the proliferation of vascular endothelial cells (Braile et al., 2020) and dysregulation of lipid metabolism causes AS (Lin C. H. et al., 2021). Above all, these signaling pathways regulated using GLXB could be summarized into three biological processes, inflammation, endothelial growth/function, and regulation of blood lipids. Therefore, we predicted that the combination mechanisms of GLXB treatment against AS included suppressing inflammation, regulating vascular endothelial growth/function, and improving blood lipid levels.

At last, the predicted mechanisms of GLXB treatment against AS were confirmed experimentally on AS mice in this study. From the aspect of inflammation, our previous studies have established AS model in ApoE^−/−^ mice and found that GLXB treatment could inhibit the levels of IL1β and TNF-α in the liver (Xu et al., 2021) and decrease the levels of IL-1β, IL-18 in the serum of HFD-fed ApoE^−/−^ mice (Ding et al., 2022). In addition, GLXB treatment could also reduce the levels of IL6 and TNF-α in the serum of hyperlipidemia rats (Zhong et al., 2020). In this study, IL-6, IL-1β, TNF-α, ALOX5, PTGS2, and p-p38 were selected as inflammatory targets for validation. The study showed that blocking ALOX5 suppressed inflammation and immune function (Sun et al., 2019). Moreover, p-p38 is a key target of the MAPK signaling pathway (Cuadrado and Nebreda, 2010). Our results indicated that the GLXB herb pair (6 g/kg) could downregulate these inflammatory targets. In addition, the expressions of VEGFA, eNOS, p-AKT, VCAM-1, ICAM-1, and macrophage infiltration were detected to validate the effects of GLXB on vascular endothelial growth and endotheliocyte function. A report shows that eNOS disorder could cause abnormal production of NO, which may damage endothelial function and trigger AS (Hong et al., 2019). P-AKT is a key target of the PI3K/AKT signaling pathway, regulating cell proliferation (Linton et al., 2019). Moreover, endothelial cell dysfunction encompasses a constellation of various alterations, including abnormal adhesion function with elevated VCAM-1 and ICAM-1 (Gimbrone and García-Cardeña, 2016; Baker et al., 2018). Therefore, our results demonstrated that the GLXB herb pair (6 g/kg) could regulate endothelial growth-related targets and improve endotheliocyte function to alleviate AS. In addition, blood lipid levels could be regulated using the GLXB herb pair (6 g/kg), which was also more significant than using Gualou (4 g/kg) or Xiebai alone (2 g/kg). Taken together, the combination mechanisms of GLXB treatment against AS were verified *in vivo*, including suppressing inflammation, regulating vascular endothelial growth/function, and improving blood lipid levels.

## 5 Conclusion

In summary, the GLXB herb pair displayed a more significant effect on treating AS than Gualou or Xiebai alone. In addition, the combination mechanisms of the GLXB herb pair were elucidated in terms of components, targets, and signaling pathways, which may be related to suppressing inflammation, regulating vascular endothelial growth/function, and improving blood lipid levels.

## Data Availability

The original contributions presented in the study are included in the article/supplementary material. Further inquiries can be directed to the corresponding authors.
